# 2-Chloro-*N*-(2-chloro­benzo­yl)-*N*-(2-ethyl-4-oxo-3,4-di­hydro­quinazolin-3-yl)benzamide

**DOI:** 10.1107/S1600536814006035

**Published:** 2014-03-29

**Authors:** Oladapo Bakare, Candice Thompson, Yakini Brandy, Ray J. Butcher

**Affiliations:** a Department of Chemistry, Howard University, 525 College Street NW, Washington, DC 20059, USA

## Abstract

In the title compound, C_24_H_17_Cl_2_N_3_O_3_, the quinazolinone ring system is close to planar (r.m.s. deviation = 0.0132 Å), with the imide unit almost perpendicular to it, subtending a dihedral angle of 89.1 (1)°. However, the imide unit itself is not planar, the dihedral angle between the two O=C—N components being 34.6 (1)°. The dihedral angle between the two chlorobenzene rings is 40.50 (7)°, while the angles between these rings and the imide moiety are 54.6 (1) and 58.2 (1)°, respectively. The dihedral angles between the 2-chloro­phenyl rings and the quinazolinone ring system are 48.77 (5) and 32.92 (7)° for rings *A* and *B*, respectively. In the crystal, weak C—H⋯O inter­actions link the mol­ecules into a three-dimensional array.

## Related literature   

For the synthesis and biological evaluation of some imido-substituted 1,4-naphtho­quinone derivatives, see: Bakare *et al.* (2003 ▶); Berhe *et al.* (2008 ▶); Brandy *et al.* (2013 ▶); Khraiwesh *et al.* (2012 ▶). For similar X-ray structures, see: Akinboye *et al.* (2009*a* ▶,*b* ▶); Brandy *et al.* (2012 ▶).
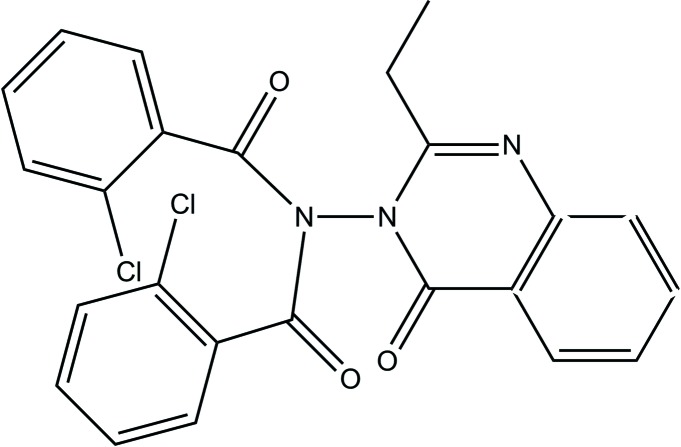


## Experimental   

### 

#### Crystal data  

C_24_H_17_Cl_2_N_3_O_3_
*M* *_r_* = 466.31
Orthorhombic, 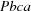

*a* = 17.2597 (6) Å
*b* = 13.5463 (4) Å
*c* = 18.9683 (7) Å
*V* = 4434.9 (3) Å^3^
*Z* = 8
Mo *K*α radiation
μ = 0.33 mm^−1^
*T* = 200 K
0.52 × 0.18 × 0.15 mm

#### Data collection  

Oxford Diffraction Gemini diffractometer
Absorption correction: multi-scan (*CrysAlis RED*; Agilent, 2012 ▶) *T*_min_ = 0.935, *T*_max_ = 1.000
32371 measured reflections
7466 independent reflections
2351 reflections with *I* > 2σ(*I*)
*R*_int_ = 0.123

#### Refinement  

*R*[*F* ^2^ > 2σ(*F* ^2^)] = 0.044
*wR*(*F* ^2^) = 0.079
*S* = 0.77
7466 reflections
290 parameters
H-atom parameters constrained
Δρ_max_ = 0.21 e Å^−3^
Δρ_min_ = −0.24 e Å^−3^

### 

Data collection: *CrysAlis CCD* (Agilent, 2012 ▶); cell refinement: *CrysAlis RED* (Agilent, 2012 ▶); data reduction: *CrysAlis RED*; program(s) used to solve structure: *SHELXS97* (Sheldrick, 2008 ▶); program(s) used to refine structure: *SHELXL97* (Sheldrick, 2008 ▶); molecular graphics: *SHELXTL* (Sheldrick, 2008 ▶); software used to prepare material for publication: *SHELXTL*.

## Supplementary Material

Crystal structure: contains datablock(s) I. DOI: 10.1107/S1600536814006035/bt6969sup1.cif

Structure factors: contains datablock(s) I. DOI: 10.1107/S1600536814006035/bt6969Isup2.hkl

Supporting information file. DOI: 10.1107/S1600536814006035/bt6969Isup3.cml

CCDC reference: 992424

Additional supporting information:  crystallographic information; 3D view; checkCIF report

## Figures and Tables

**Table 1:** Hydrogen-bond geometry (Å, °)

*D*—H⋯*A*	*D*—H	H⋯*A*	*D*⋯*A*	*D*—H⋯*A*
C7*A*—H7*AA*⋯O^i^	0.95	2.43	3.143 (2)	132
C4*B*—H4*BA*⋯O1*A* ^ii^	0.95	2.35	3.211 (2)	151
C6—H6*A*⋯O1*A* ^iii^	0.95	2.58	3.377 (2)	142

Symmetry codes: (i) 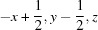; (ii) 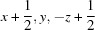; (iii) 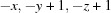.

**Fig. 1.**
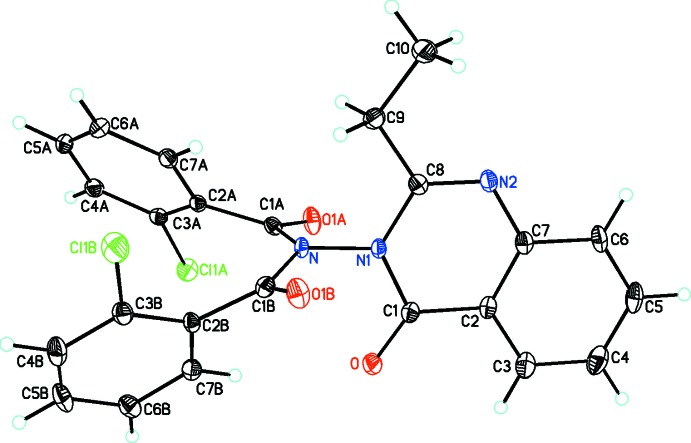
ORTEP diagram of the title compound showing the atom numbering scheme. Atomic displacement parameters are drawn at the 30% probability level.

**Fig. 2.**
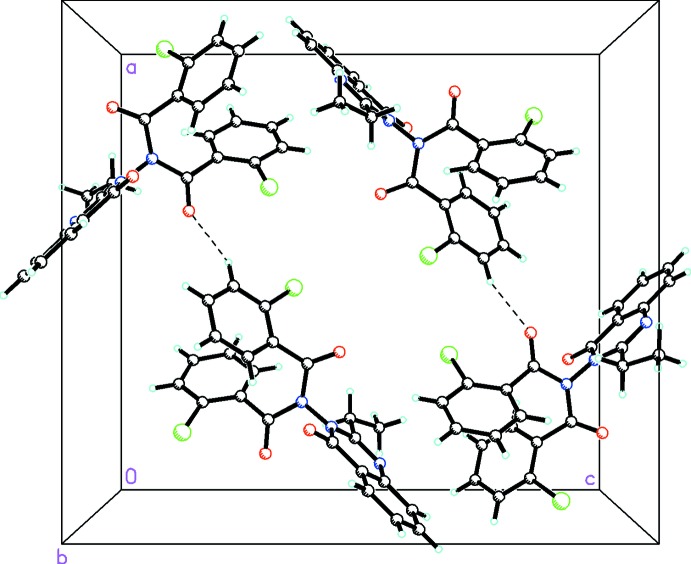
Packing diagram for the complex viewed along the b axis. C—H···O interactions shown by dashed lines.

## supplementary crystallographic information

### 1. Chemical context

We have been involved in the synthesis and biological evaluation of some imido-substituted 1,4-naphtho­quinone derivatives (Bakare *et al.*, 2003; Berhe *et al.*, 2008; Brandy *et al.*, 2013). These compounds have been shown to exhibit some anti-cancer (Bakare *et al.*, 2003; Berhe *et al.*, 2008) and anti-trypanosomal (Khraiwesh *et al.*, (2012) activities. In an attempt to study the effect of replacing the naphtho­quinone scaffold with quinazolinone, on biological activities of this class of compounds, we have synthesized and structurally characterized some imido-substituted quinazolinone derivatives (Akinboye *et al.*, 2009*a*, 2009*b*). We here report the crystal structure properties of 2-chloro-N-(2-chloro-benzoyl)-N-(2-ethyl-4-oxo-4H-quinazolin-3-yl)-benzamide. Weak C—H···O inter­actions link the molecules into a 3-D array.

### 2. Structural commentary

In the title compound, C_24_H_17_Cl_2_N_3_O_3_, the quinazolinone ring is planar with the imide moiety (O1A C1A N C1B O1B) almost perpendicular with a dihedral angle of 89.1 (1)°. However, the imide moiety itself is not strictly planar. The dihedral angle between the two components (O1A C1A N and O1B C1B N) is 34.6 (1)°. The dihedral angle between the two 2-chloro­phenyl rings is 40.50 (7)° while the angles between these and the imide moiety are 54.6 (1)° and 58.2 (1)° for rings A and B respectively. The dihedral angles between the 2-chloro­phenyl rings and the quinazolinone ring are 48.77 (5)° and 32.92 (7)° for A and B respectively. Weak C—H···O inter­actions link the molecules into a 3-D array.

### 3. Supra­molecular features

Weak C—H···O inter­actions link the molecules into a 3-D array.

### 4. Database survey

For the synthesis and biological evaluation of some imido-substituted 1,4-naphtho­quinone derivatives, see; Bakare *et al.* (2003); Berhe *et al.* (2008); Brandy *et al.* (2013); Khraiwesh *et al.* (2012). For similar x-ray structures see Akinboye *et al.* (2009*a*, 2009*b*); Brandy *et al.* (2012).

### 5. Synthesis and crystallization

To a solution of 3-amino-2-ethyl-4(3H) quinazolinone (186 mg) in tetra­hydro­furan (15 mL) was added NaH (70.7 mg) and the mixture stirred at room temperature for 15 min. 2-Chloro-benzoyl chloride (0.273 mL) was added drop wise and the resulting mixture stirred at room temperature for 20 hr. The reaction mixture was poured into a mixture of ice (10 g) and water (10 mL) and then extracted with di­chloro­methane (25 mL). The organic layer was washed with water (3X20 mL), saturated sodium chloride solution (20 mL), dried over anhydrous MgSO_4_ and the solvent removed in vacuo to give a white solid. The crude white solid was dissolved in hot ethanol:water mixture (2:3, 5 mL) from which the title compound crystallized at room temperature after 6 days.

### 6. Refinement

H atoms were placed in geometrically idealized positions with a C—H distances of 0.95 and 0.99 Å *U*_iso_(H) = 1.2*U*_eq_(C) and 0.98 Å for CH_3_ [*U*_iso_(H) = 1.5*U*_eq_(C)].

### Crystal data

**Table:**

C_24_H_17_Cl_2_N_3_O_3_	*F*(000) = 1920
*M**_r_* = 466.31	*D*_x_ = 1.397 Mg m^−^^3^
Orthorhombic, *P**b**c**a*	Mo *K*α radiation, λ = 0.71073 Å
Hall symbol: -P 2ac 2ab	Cell parameters from 4113 reflections
*a* = 17.2597 (6) Å	θ = 4.6–32.5°
*b* = 13.5463 (4) Å	µ = 0.33 mm^−^^1^
*c* = 18.9683 (7) Å	*T* = 200 K
*V* = 4434.9 (3) Å^3^	Needle, colourless
*Z* = 8	0.52 × 0.18 × 0.15 mm

### Data collection

**Table:**

Oxford Diffraction Gemini diffractometer	7466 independent reflections
Radiation source: fine-focus sealed tube	2351 reflections with *I* > 2σ(*I*)
Graphite monochromator	*R*_int_ = 0.123
Detector resolution: 10.5081 pixels mm^-1^	θ_max_ = 32.6°, θ_min_ = 4.6°
φ and ω scans	*h* = −26→25
Absorption correction: multi-scan (*CrysAlis RED*; Agilent, 2012)	*k* = −19→20
*T*_min_ = 0.935, *T*_max_ = 1.000	*l* = −25→28
32371 measured reflections	

### Refinement

**Table:**

Refinement on *F*^2^	Primary atom site location: structure-invariant direct methods
Least-squares matrix: full	Secondary atom site location: difference Fourier map
*R*[*F*^2^ > 2σ(*F*^2^)] = 0.044	Hydrogen site location: inferred from neighbouring sites
*wR*(*F*^2^) = 0.079	H-atom parameters constrained
*S* = 0.77	*w* = 1/[σ^2^(*F*_o_^2^) + (0.0239*P*)^2^] where *P* = (*F*_o_^2^ + 2*F*_c_^2^)/3
7466 reflections	(Δ/σ)_max_ = 0.001
290 parameters	Δρ_max_ = 0.21 e Å^−^^3^
0 restraints	Δρ_min_ = −0.24 e Å^−^^3^

### Special details

**Table:**

Geometry. All e.s.d.'s (except the e.s.d. in the dihedral angle between two l.s. planes) are estimated using the full covariance matrix. The cell e.s.d.'s are taken into account individually in the estimation of e.s.d.'s in distances, angles and torsion angles; correlations between e.s.d.'s in cell parameters are only used when they are defined by crystal symmetry. An approximate (isotropic) treatment of cell e.s.d.'s is used for estimating e.s.d.'s involving l.s. planes.
Refinement. Refinement of *F*^2^ against ALL reflections. The weighted *R*-factor *wR* and goodness of fit *S* are based on *F*^2^, conventional *R*-factors *R* are based on *F*, with *F* set to zero for negative *F*^2^. The threshold expression of *F*^2^ > σ(*F*^2^) is used only for calculating *R*-factors(gt) *etc*. and is not relevant to the choice of reflections for refinement. *R*-factors based on *F*^2^ are statistically about twice as large as those based on *F*, and *R*- factors based on ALL data will be even larger.

### Fractional atomic coordinates and isotropic or equivalent isotropic displacement parameters (Å^2^)

**Table:**

	*x*	*y*	*z*	*U*_iso_*/*U*_eq_	
Cl1A	0.17309 (3)	0.55900 (4)	0.16919 (3)	0.04342 (14)	
Cl1B	0.46697 (3)	0.43992 (4)	0.37319 (3)	0.05512 (17)	
O	0.18853 (8)	0.70694 (9)	0.40899 (7)	0.0423 (4)	
O1A	0.12604 (7)	0.51488 (9)	0.32178 (7)	0.0417 (4)	
O1B	0.33657 (7)	0.55297 (10)	0.46096 (7)	0.0433 (3)	
N	0.23311 (8)	0.52344 (9)	0.39013 (7)	0.0249 (4)	
N1	0.18318 (8)	0.54745 (10)	0.44570 (7)	0.0265 (3)	
N2	0.10350 (9)	0.49031 (11)	0.53679 (8)	0.0328 (4)	
C1	0.15976 (11)	0.64646 (14)	0.44890 (10)	0.0297 (5)	
C2	0.10246 (10)	0.66490 (14)	0.50310 (10)	0.0315 (5)	
C3	0.07421 (12)	0.76030 (15)	0.51353 (11)	0.0447 (6)	
H3A	0.0929	0.8133	0.4854	0.054*	
C4	0.01927 (13)	0.77749 (17)	0.56451 (12)	0.0550 (7)	
H4A	0.0000	0.8424	0.5719	0.066*	
C5	−0.00805 (12)	0.69966 (18)	0.60521 (12)	0.0516 (6)	
H5A	−0.0460	0.7118	0.6404	0.062*	
C6	0.01906 (11)	0.60552 (16)	0.59530 (10)	0.0415 (5)	
H6A	−0.0004	0.5531	0.6234	0.050*	
C7	0.07533 (10)	0.58646 (14)	0.54378 (10)	0.0311 (5)	
C8	0.15506 (10)	0.47290 (12)	0.48974 (10)	0.0283 (5)	
C9	0.18893 (12)	0.37229 (12)	0.47998 (10)	0.0376 (5)	
H9A	0.2461	0.3775	0.4800	0.045*	
H9B	0.1729	0.3463	0.4334	0.045*	
C10	0.16416 (13)	0.29949 (14)	0.53710 (10)	0.0543 (6)	
H10A	0.1871	0.2347	0.5275	0.081*	
H10B	0.1076	0.2938	0.5374	0.081*	
H10C	0.1819	0.3232	0.5831	0.081*	
C1A	0.19483 (11)	0.49986 (12)	0.32637 (10)	0.0269 (4)	
C2A	0.24009 (10)	0.44610 (13)	0.27213 (9)	0.0248 (4)	
C3A	0.22865 (10)	0.46128 (13)	0.20026 (10)	0.0295 (4)	
C4A	0.26354 (11)	0.40133 (13)	0.15073 (10)	0.0359 (5)	
H4AA	0.2551	0.4123	0.1019	0.043*	
C5A	0.31076 (12)	0.32527 (13)	0.17292 (11)	0.0401 (5)	
H5AA	0.3342	0.2831	0.1391	0.048*	
C6A	0.32422 (11)	0.30990 (13)	0.24376 (11)	0.0372 (5)	
H6AA	0.3577	0.2583	0.2586	0.045*	
C7A	0.28880 (10)	0.36974 (13)	0.29274 (10)	0.0304 (5)	
H7AA	0.2978	0.3586	0.3415	0.037*	
C1B	0.31255 (10)	0.54950 (13)	0.40145 (10)	0.0292 (4)	
C2B	0.35792 (10)	0.57646 (12)	0.33781 (9)	0.0258 (4)	
C3B	0.42944 (10)	0.53492 (13)	0.32191 (10)	0.0334 (5)	
C4B	0.47126 (11)	0.56635 (16)	0.26462 (11)	0.0447 (5)	
H4BA	0.5197	0.5365	0.2539	0.054*	
C5B	0.44293 (12)	0.64119 (16)	0.22275 (11)	0.0481 (6)	
H5BA	0.4719	0.6628	0.1830	0.058*	
C6B	0.37257 (12)	0.68506 (14)	0.23818 (11)	0.0412 (5)	
H6BA	0.3535	0.7375	0.2098	0.049*	
C7B	0.33058 (11)	0.65183 (12)	0.29506 (10)	0.0329 (5)	
H7BA	0.2818	0.6811	0.3053	0.040*	

### Atomic displacement parameters (Å^2^)

**Table:**

	*U*^11^	*U*^22^	*U*^33^	*U*^12^	*U*^13^	*U*^23^
Cl1A	0.0517 (3)	0.0467 (3)	0.0319 (3)	0.0063 (3)	−0.0019 (3)	0.0055 (2)
Cl1B	0.0382 (3)	0.0594 (4)	0.0677 (4)	0.0131 (3)	0.0015 (3)	0.0122 (3)
O	0.0556 (9)	0.0345 (8)	0.0369 (9)	0.0039 (7)	0.0148 (8)	0.0053 (6)
O1A	0.0225 (7)	0.0719 (10)	0.0307 (9)	0.0020 (7)	−0.0006 (7)	−0.0031 (7)
O1B	0.0359 (8)	0.0682 (9)	0.0259 (8)	−0.0045 (7)	−0.0048 (7)	−0.0015 (7)
N	0.0235 (8)	0.0331 (9)	0.0181 (9)	0.0008 (7)	0.0038 (8)	−0.0038 (6)
N1	0.0281 (8)	0.0300 (9)	0.0212 (9)	0.0017 (8)	0.0064 (8)	−0.0011 (7)
N2	0.0319 (9)	0.0397 (11)	0.0267 (10)	−0.0036 (8)	0.0064 (9)	−0.0023 (7)
C1	0.0317 (11)	0.0318 (12)	0.0256 (12)	0.0047 (10)	0.0003 (10)	−0.0012 (9)
C2	0.0313 (11)	0.0389 (12)	0.0245 (12)	0.0057 (10)	0.0014 (10)	−0.0037 (9)
C3	0.0487 (13)	0.0504 (15)	0.0348 (14)	0.0140 (12)	0.0050 (12)	0.0018 (10)
C4	0.0586 (16)	0.0568 (16)	0.0495 (17)	0.0246 (13)	0.0052 (14)	−0.0088 (12)
C5	0.0374 (13)	0.0756 (18)	0.0417 (15)	0.0153 (13)	0.0119 (12)	−0.0125 (13)
C6	0.0328 (12)	0.0632 (15)	0.0286 (13)	−0.0028 (11)	0.0113 (11)	−0.0032 (10)
C7	0.0254 (10)	0.0432 (13)	0.0247 (12)	−0.0008 (10)	−0.0017 (10)	−0.0032 (9)
C8	0.0280 (11)	0.0349 (12)	0.0221 (11)	−0.0034 (9)	−0.0043 (10)	−0.0001 (8)
C9	0.0480 (13)	0.0309 (12)	0.0339 (13)	0.0026 (10)	0.0028 (11)	−0.0007 (9)
C10	0.0830 (17)	0.0398 (13)	0.0401 (14)	0.0017 (13)	0.0111 (14)	0.0063 (10)
C1A	0.0277 (10)	0.0312 (10)	0.0219 (11)	−0.0047 (9)	−0.0003 (10)	0.0020 (8)
C2A	0.0234 (9)	0.0266 (10)	0.0244 (11)	−0.0064 (9)	0.0018 (9)	−0.0043 (8)
C3A	0.0286 (10)	0.0283 (11)	0.0315 (12)	−0.0050 (9)	0.0016 (10)	−0.0017 (8)
C4A	0.0502 (13)	0.0324 (12)	0.0252 (12)	−0.0064 (11)	0.0074 (11)	−0.0006 (9)
C5A	0.0513 (13)	0.0322 (12)	0.0368 (14)	−0.0057 (11)	0.0184 (12)	−0.0101 (9)
C6A	0.0400 (12)	0.0281 (11)	0.0434 (14)	0.0032 (10)	0.0061 (12)	−0.0030 (10)
C7A	0.0319 (11)	0.0320 (12)	0.0273 (12)	−0.0056 (10)	0.0032 (10)	−0.0012 (9)
C1B	0.0260 (10)	0.0317 (11)	0.0298 (12)	0.0034 (9)	−0.0023 (10)	−0.0033 (9)
C2B	0.0219 (9)	0.0336 (11)	0.0219 (11)	−0.0045 (9)	0.0013 (9)	−0.0040 (8)
C3B	0.0257 (10)	0.0394 (12)	0.0351 (13)	−0.0002 (9)	−0.0009 (10)	−0.0004 (9)
C4B	0.0265 (11)	0.0655 (15)	0.0421 (14)	0.0007 (12)	0.0049 (11)	−0.0075 (12)
C5B	0.0379 (13)	0.0743 (17)	0.0321 (14)	−0.0111 (13)	0.0111 (12)	0.0042 (12)
C6B	0.0411 (12)	0.0477 (13)	0.0346 (14)	−0.0070 (11)	0.0010 (12)	0.0054 (10)
C7B	0.0282 (10)	0.0385 (12)	0.0321 (12)	−0.0023 (10)	−0.0009 (10)	−0.0059 (9)

### Geometric parameters (Å, º)

**Table:**

Cl1A—C3A	1.7376 (18)	C9—H9B	0.9900
Cl1B—C3B	1.7385 (19)	C10—H10A	0.9800
O—C1	1.2211 (19)	C10—H10B	0.9800
O1A—C1A	1.208 (2)	C10—H10C	0.9800
O1B—C1B	1.2034 (19)	C1A—C2A	1.483 (2)
N—N1	1.3999 (17)	C2A—C7A	1.389 (2)
N—C1A	1.415 (2)	C2A—C3A	1.393 (2)
N—C1B	1.432 (2)	C3A—C4A	1.380 (2)
N1—C8	1.398 (2)	C4A—C5A	1.379 (2)
N1—C1	1.402 (2)	C4A—H4AA	0.9500
N2—C8	1.282 (2)	C5A—C6A	1.379 (3)
N2—C7	1.397 (2)	C5A—H5AA	0.9500
C1—C2	1.448 (2)	C6A—C7A	1.376 (2)
C2—C7	1.394 (2)	C6A—H6AA	0.9500
C2—C3	1.395 (2)	C7A—H7AA	0.9500
C3—C4	1.374 (3)	C1B—C2B	1.485 (2)
C3—H3A	0.9500	C2B—C7B	1.387 (2)
C4—C5	1.389 (3)	C2B—C3B	1.390 (2)
C4—H4A	0.9500	C3B—C4B	1.372 (2)
C5—C6	1.371 (3)	C4B—C5B	1.378 (3)
C5—H5A	0.9500	C4B—H4BA	0.9500
C6—C7	1.402 (2)	C5B—C6B	1.383 (3)
C6—H6A	0.9500	C5B—H5BA	0.9500
C8—C9	1.494 (2)	C6B—C7B	1.376 (2)
C9—C10	1.526 (2)	C6B—H6BA	0.9500
C9—H9A	0.9900	C7B—H7BA	0.9500
			
N1—N—C1A	114.12 (13)	O1A—C1A—N	118.87 (17)
N1—N—C1B	114.78 (13)	O1A—C1A—C2A	123.41 (18)
C1A—N—C1B	129.13 (15)	N—C1A—C2A	117.25 (15)
C8—N1—N	119.73 (14)	C7A—C2A—C3A	118.12 (16)
C8—N1—C1	124.42 (15)	C7A—C2A—C1A	119.29 (16)
N—N1—C1	115.61 (13)	C3A—C2A—C1A	122.13 (17)
C8—N2—C7	118.64 (16)	C4A—C3A—C2A	121.17 (17)
O—C1—N1	119.86 (17)	C4A—C3A—Cl1A	117.27 (15)
O—C1—C2	127.02 (17)	C2A—C3A—Cl1A	121.51 (14)
N1—C1—C2	113.11 (16)	C5A—C4A—C3A	119.31 (18)
C7—C2—C3	120.68 (18)	C5A—C4A—H4AA	120.3
C7—C2—C1	119.39 (17)	C3A—C4A—H4AA	120.3
C3—C2—C1	119.93 (18)	C6A—C5A—C4A	120.63 (18)
C4—C3—C2	119.8 (2)	C6A—C5A—H5AA	119.7
C4—C3—H3A	120.1	C4A—C5A—H5AA	119.7
C2—C3—H3A	120.1	C7A—C6A—C5A	119.60 (18)
C3—C4—C5	119.8 (2)	C7A—C6A—H6AA	120.2
C3—C4—H4A	120.1	C5A—C6A—H6AA	120.2
C5—C4—H4A	120.1	C6A—C7A—C2A	121.16 (18)
C6—C5—C4	120.9 (2)	C6A—C7A—H7AA	119.4
C6—C5—H5A	119.5	C2A—C7A—H7AA	119.4
C4—C5—H5A	119.5	O1B—C1B—N	118.70 (17)
C5—C6—C7	120.2 (2)	O1B—C1B—C2B	124.85 (16)
C5—C6—H6A	119.9	N—C1B—C2B	116.34 (16)
C7—C6—H6A	119.9	C7B—C2B—C3B	118.26 (17)
C2—C7—N2	122.77 (17)	C7B—C2B—C1B	118.50 (16)
C2—C7—C6	118.56 (18)	C3B—C2B—C1B	123.05 (17)
N2—C7—C6	118.64 (18)	C4B—C3B—C2B	120.90 (18)
N2—C8—N1	121.61 (16)	C4B—C3B—Cl1B	118.48 (15)
N2—C8—C9	121.70 (17)	C2B—C3B—Cl1B	120.61 (15)
N1—C8—C9	116.69 (16)	C3B—C4B—C5B	119.87 (19)
C8—C9—C10	113.07 (16)	C3B—C4B—H4BA	120.1
C8—C9—H9A	109.0	C5B—C4B—H4BA	120.1
C10—C9—H9A	109.0	C4B—C5B—C6B	120.4 (2)
C8—C9—H9B	109.0	C4B—C5B—H5BA	119.8
C10—C9—H9B	109.0	C6B—C5B—H5BA	119.8
H9A—C9—H9B	107.8	C7B—C6B—C5B	119.20 (19)
C9—C10—H10A	109.5	C7B—C6B—H6BA	120.4
C9—C10—H10B	109.5	C5B—C6B—H6BA	120.4
H10A—C10—H10B	109.5	C6B—C7B—C2B	121.35 (18)
C9—C10—H10C	109.5	C6B—C7B—H7BA	119.3
H10A—C10—H10C	109.5	C2B—C7B—H7BA	119.3
H10B—C10—H10C	109.5		
			
C1A—N—N1—C8	88.76 (18)	C1B—N—C1A—C2A	34.5 (2)
C1B—N—N1—C8	−105.67 (17)	O1A—C1A—C2A—C7A	−130.35 (19)
C1A—N—N1—C1	−85.93 (17)	N—C1A—C2A—C7A	41.7 (2)
C1B—N—N1—C1	79.63 (18)	O1A—C1A—C2A—C3A	41.7 (3)
C8—N1—C1—O	178.75 (16)	N—C1A—C2A—C3A	−146.27 (16)
N—N1—C1—O	−6.8 (2)	C7A—C2A—C3A—C4A	1.1 (2)
C8—N1—C1—C2	−0.1 (2)	C1A—C2A—C3A—C4A	−171.08 (16)
N—N1—C1—C2	174.35 (14)	C7A—C2A—C3A—Cl1A	−176.41 (12)
O—C1—C2—C7	179.50 (19)	C1A—C2A—C3A—Cl1A	11.4 (2)
N1—C1—C2—C7	−1.8 (2)	C2A—C3A—C4A—C5A	−0.2 (3)
O—C1—C2—C3	0.3 (3)	Cl1A—C3A—C4A—C5A	177.38 (14)
N1—C1—C2—C3	179.05 (17)	C3A—C4A—C5A—C6A	−1.1 (3)
C7—C2—C3—C4	0.2 (3)	C4A—C5A—C6A—C7A	1.4 (3)
C1—C2—C3—C4	179.35 (18)	C5A—C6A—C7A—C2A	−0.5 (3)
C2—C3—C4—C5	−0.2 (3)	C3A—C2A—C7A—C6A	−0.7 (2)
C3—C4—C5—C6	0.0 (3)	C1A—C2A—C7A—C6A	171.68 (16)
C4—C5—C6—C7	0.2 (3)	N1—N—C1B—O1B	27.8 (2)
C3—C2—C7—N2	−177.99 (17)	C1A—N—C1B—O1B	−169.29 (16)
C1—C2—C7—N2	2.9 (3)	N1—N—C1B—C2B	−148.52 (14)
C3—C2—C7—C6	0.0 (3)	C1A—N—C1B—C2B	14.4 (2)
C1—C2—C7—C6	−179.09 (16)	O1B—C1B—C2B—C7B	−120.3 (2)
C8—N2—C7—C2	−1.9 (3)	N—C1B—C2B—C7B	55.7 (2)
C8—N2—C7—C6	−179.90 (16)	O1B—C1B—C2B—C3B	54.6 (3)
C5—C6—C7—C2	−0.3 (3)	N—C1B—C2B—C3B	−129.34 (18)
C5—C6—C7—N2	177.84 (18)	C7B—C2B—C3B—C4B	−1.2 (3)
C7—N2—C8—N1	−0.1 (2)	C1B—C2B—C3B—C4B	−176.20 (17)
C7—N2—C8—C9	178.94 (16)	C7B—C2B—C3B—Cl1B	−179.81 (13)
N—N1—C8—N2	−173.11 (15)	C1B—C2B—C3B—Cl1B	5.2 (2)
C1—N1—C8—N2	1.1 (3)	C2B—C3B—C4B—C5B	1.1 (3)
N—N1—C8—C9	7.8 (2)	Cl1B—C3B—C4B—C5B	179.71 (15)
C1—N1—C8—C9	−178.03 (16)	C3B—C4B—C5B—C6B	0.1 (3)
N2—C8—C9—C10	−7.8 (3)	C4B—C5B—C6B—C7B	−1.2 (3)
N1—C8—C9—C10	171.29 (16)	C5B—C6B—C7B—C2B	1.0 (3)
N1—N—C1A—O1A	9.9 (2)	C3B—C2B—C7B—C6B	0.1 (3)
C1B—N—C1A—O1A	−153.11 (17)	C1B—C2B—C7B—C6B	175.35 (16)
N1—N—C1A—C2A	−162.47 (14)		

### Hydrogen-bond geometry (Å, º)

**Table:**

*D*—H···*A*	*D*—H	H···*A*	*D*···*A*	*D*—H···*A*
C7*A*—H7*AA*···O^i^	0.95	2.43	3.143 (2)	132
C4*B*—H4*BA*···O1*A*^ii^	0.95	2.35	3.211 (2)	151
C6—H6*A*···O1*A*^iii^	0.95	2.58	3.377 (2)	142

Symmetry codes: (i) −*x*+1/2, *y*−1/2, *z*; (ii) *x*+1/2, *y*, −*z*+1/2; (iii) −*x*, −*y*+1, −*z*+1.
